# Data sharing in circadian rhythm and mental health research: current status, challenges, recommendations and future directions.

**DOI:** 10.1136/bmjment-2024-301333

**Published:** 2024-12-22

**Authors:** Haya Deeb, Tomasz Zieliński, Andrew J Millar

**Affiliations:** 1 Centre for Engineering Biology and School of Biological Sciences, University of Edinburgh, Edinburgh, UK

**Keywords:** data interpretation, statistical, sleep, psychiatry

## Abstract

Data sharing is a cornerstone of modern scientific research, playing a critical role in fostering greater collaboration, enhancing reproducibility, transparency and efficiency of scientific discoveries, and integrating diverse data sources. In circadian rhythm research, data sharing is particularly important due to the complexity and heterogeneity of the data, which includes molecular profiles, physiological measurements, clinical data and sensor-based data. UK research funders, such as Medical Research Council, Wellcome Trust and UK Research and Innovation, have established data-sharing policies to promote open science and enhance research transparency. Despite these policies, a recent assessment within the UK Circadian Mental Health Network (CMHN), which incorporates an analysis of publications from several countries, revealed that data sharing remains limited. Significant challenges including data complexity, privacy and ethical considerations, technical issues and entrenched academic culture are major barriers to progress. This perspective article highlights the current state of data sharing in circadian and mental health research, identifies key obstacles and compares these practices with broader trends. We also provide insights from principal investigators within the CMHN on the reasons for limited data sharing. To address these challenges, researchers can foster a culture of openness by seeking training, planning ahead in ethics processes and data management plans and using data outputs in research assessment. We outline CMHN’s future plans to deliver training on Findable, Accessible, Interoperable, Reusable principles, offer data curation services and provide ethical guidelines. By adopting these strategies, we aim to improve data-sharing practices, ultimately advancing our understanding of circadian rhythms and their implications for mental health.

## Introduction

 Data sharing is a cornerstone of modern scientific research, playing a critical role in fostering greater collaboration,^1^ enhancing the reproducibility, transparency and efficiency of scientific discoveries and integrating diverse data sources.^2^ By making data openly available, researchers can validate and extend findings through multidimensional analyses, foster collaborative efforts across disciplines and ultimately accelerate scientific progress while maximising the return on research investments.

UK research funders, including the Medical Research Council (MRC), Wellcome Trust and UK Research and Innovation (UKRI), have established policies emphasising data accessibility to maximise public investment impact. The MRC and Wellcome Trust both require researchers to create a data management plan (DMP) and make data available with minimal restrictions, typically within 12 months of study completion.^3 4^ These policies also stress the use of repositories that adhere to Findable, Accessible, Interoperable, Reusable (FAIR) principles. UKRI similarly mandates open data sharing, encouraging consideration of data accessibility from the earliest stages of research design.^5^

Despite these policies, discrepancies between policy and practice might persist. Significant differences persist across disciplines in data availability and willingness to share data, even within high-impact journals that enforce strict data-sharing policies.^6^ This gap between policy and practice underscores the need for a focused examination of data sharing within specific domains. To address this, the Circadian Mental Health Network (CMHN)^7^ has conducted an assessment of data-sharing practices and challenges within the fields of circadian rhythm and mental health research.

This perspective discusses how circadian rhythm and mental health research are engaging with these data-sharing policies, comparing current practices in these fields with broader trends. We identify specific challenges and outline the roles of different stakeholders in overcoming these barriers. We conclude with targeted recommendations for researchers to enhance data-sharing practices, aiming to advance our understanding of circadian rhythms and their implications for mental health.

### Data sharing in the field of circadian rhythm and mental health

Circadian rhythms, which regulate the sleep-wake cycle and other physiological processes,^8 9^ are closely linked to various mental health conditions, including bipolar disorder, depression and are often manifested through symptoms including sleep disturbances.^8 10^ Understanding these complex interactions requires comprehensive and multifaceted datasets that span molecular profiles, physiological measurements, clinical data and sensor-based data.^11 12^ The UK CMHN was established to link mental health researchers with laboratory researchers in chronobiology and with the field of sleep research.^13^

Data sharing is particularly important in this field, because it requires costly, longitudinal studies. Some involve manual sampling with researchers working in shifts, many require automated recording equipment and some use specialised facilities where the subjects’ rhythmic environmental conditions are closely controlled. Making such data openly available can avoid duplication and optimise resource use. However, our recent assessment showed that data sharing within the CMHN remains limited.^7^ *Although the UK CMHN receives funding from domestic bodies, only 46% of the published papers within this network were supported by UK funders. All studies were conducted by principal investigators (PIs) registered within the CMHN, 60% of whom are based in the UK*.

**Figure 1:**
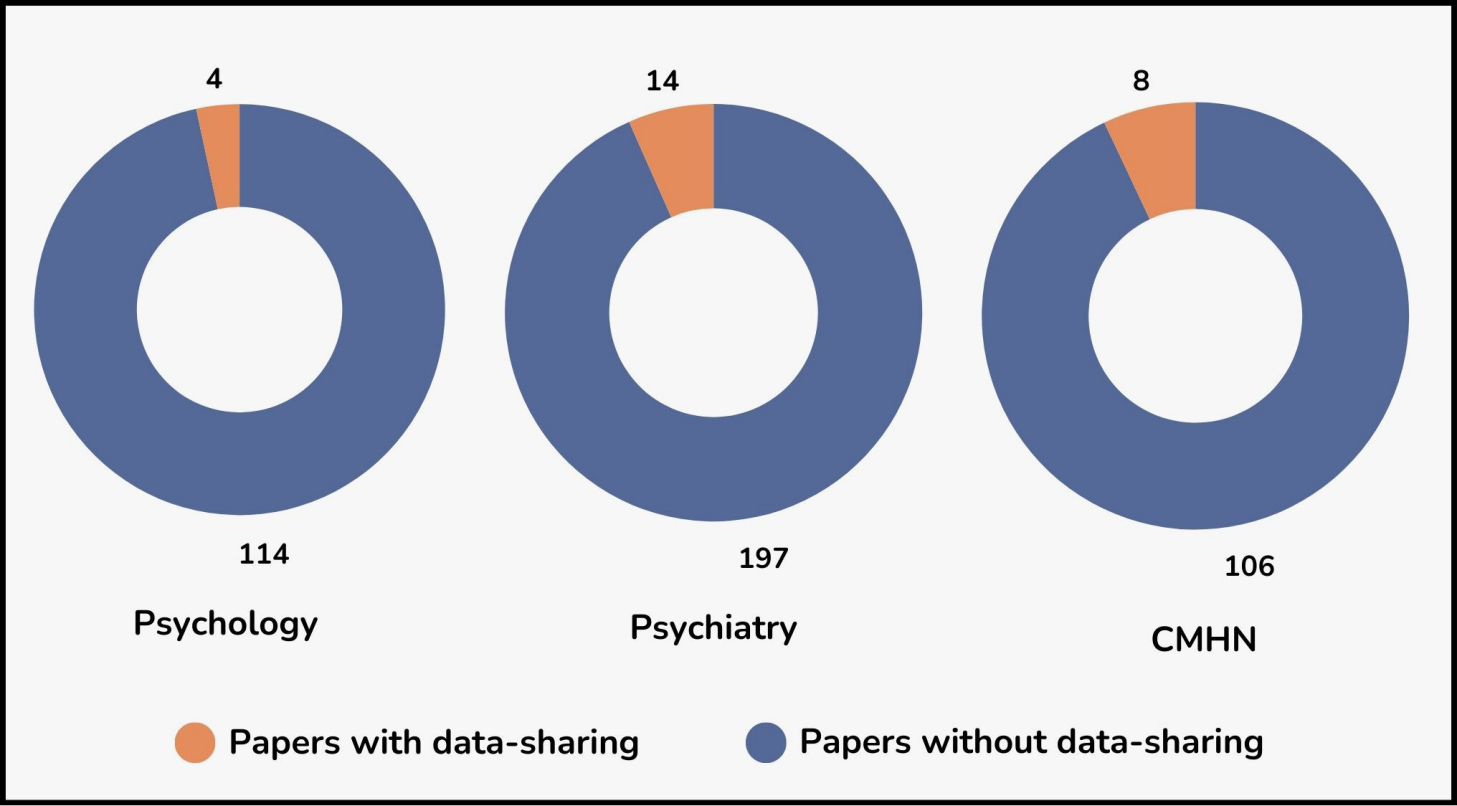
Percentage of papers sharing data in psychology (n=188, year 2014–2018, UK papers=20),^15^ psychiatry (n=211, year 2014–2018, UK papers=19)^14^ and the Circadian Mental Health Network (CMHN) research (n=114, year 2023, UK papers=53).^7^

Previous studies have assessed data-sharing practices in psychology and psychiatry publications,^14 15^ revealing that only four out of 188 psychology papers and 14 out of 211 psychiatry papers shared their data. Recently, an evaluation of the CMHN found that eight out of 114 papers shared some or all of their data^7^ (figure 1). Although these assessments span different periods (2014–2018 for psychology and psychiatry, and 2023 for the CMHN), a common trend emerges: data sharing is frequently sporadic and falls short of expectations. Moreover, this trend is also evident when examining the sharing of analysis scripts and code. In the psychology and psychiatry studies, only one paper shared its analysis script, compared with eight studies in the CMHN that shared their code.

Researchers in the field vary their methods of data sharing, with some using repositories that facilitate easy access, while others use supplementary materials, which are less findable and accessible. Among these, data sharing was most frequently observed from laboratory studies using animal models and studies involving volunteering human participants but not patients.^7^

Moreover, the interdisciplinary nature of circadian research complicates data management and sharing. While fields like biology and biomedicine, which also handle diverse data types, have shown significant improvement in data-sharing practices,^16^ circadian and mental health studies still lack the necessary data and code availability for effective collaboration and reproducibility. This lack of standardisation in data-sharing protocols further exacerbates the challenge of integrating diverse data sources from multiple disciplines.

### Challenges in sharing data in circadian mental health research

Despite the recognised benefits of data sharing, several significant challenges delay its widespread adoption in circadian mental health research.

#### Lack of technical tools

Technical issues are a significant barrier to effective data sharing. Many research institutions, especially smaller ones, face challenges with inadequate infrastructure for data storage and management, which is crucial for public sharing. One PI within the CMHN commented on the lack of suitable and specific repositories: ‘*There are too many different data repository options, and labs lack resources to develop and maintain their own. Well-curated metadata is also essential for sharing long-term activity data too’*. The lack of interoperable databases and standardised data formats further increases this problem, leading to difficulties in integrating data from diverse sources. Previous evaluations have pointed out that many researchers rely on supplementary materials for data sharing, which often leads to reduced accessibility and visuality of the data.^6^ Establishing dedicated repositories for specific types of data could enhance data-sharing practices and promote more effective collaboration within the research community.

#### Difficulty in sharing and linkage of multisource data

Another major challenge in circadian mental health research is the diversity and complexity of data sources. Circadian research encompasses a broad spectrum of data types, including molecular profiles, physiological measurements and sensor-based data, each requiring specialised knowledge and tools for accurate handling and interpretation.^12^ The recent CMHN assessment project illustrated this complexity, with 65 out of 114 studies employing multiple data collection methods.^7^ The range of data types complicates integration, linkage and standardisation. To maintain consistency across research initiatives, these complexities demand the continual development and revision of comprehensive data management protocols. This dynamic environment creates a significant challenge in forming standardised, unified datasets or metadata that facilitate effective data sharing and collaborative analysis within the field.

#### Ethical and privacy challenges

Mental health data are highly sensitive, raising concerns about patient confidentiality and data security.^17^ To better understand these issues, we reached out to PIs within the CMHN to provide insights into why data were not shared in specific studies. One PI noted, “*We did not have consent from these participants to share their data. This was a small sample size, so there was a higher chance for reidentification. We also felt that the NHS ethics committee would not approve data sharing given the small sample size and clinical sample*”. This highlights the necessity of incorporating data-sharing plans in funding applications and obtaining explicit informed consent, ensuring adherence to ethical guidelines and patient confidentiality.

Moreover, inconsistencies in data privacy regulations across institutional, national and international boundaries complicate cross-border data sharing.^18^ The complexity is further compounded in cases of secondary analysis of datasets, such as those from the UK BioBank or NHS patient data, where researchers do not own the data and cannot directly decide on data sharing, as these decisions are governed by the policies of the custodian organisations. The practical execution of data sharing in mental health research, notably the anonymisation process and database maintenance, involves substantial time and resources, underscoring the pressing need for adequate institutional and financial support to address these complexities effectively.

#### Need for clear academic and publishing policies

Researchers may be reluctant to share their data due to fears of being scooped, losing competitive advantage or receiving inadequate recognition for their contributions.^19^ One PI respondent pointed out, ‘*There are issues of consent/resources and prior data-sharing agreements’*. Additionally, One of the PIs mentioned, “*Clearer guidelines on data sharing are needed. I think journals will need to require it for there to be substantial change*”. Additionally, the current metrics for academic success, which prioritise publications and citations over data sharing and collaboration, further disincentivise open data practices.

### Recommendations for improving data-sharing practices

Gaining the benefits associated with data sharing in circadian mental health research will require comprehensive strategies that address the needs of various stakeholders, including researchers, institutions and funding bodies. Promoting a culture of openness and collaboration is crucial, supported by policies and infrastructure that facilitate data sharing while protecting participant privacy. Implementing the FAIR data principles can enhance the overall quality and usability of shared data. Additionally, developing incentives for data sharing, such as recognition in academic evaluations and funding opportunities specifically for data-sharing initiatives, could encourage more researchers to embrace open data practices. One of the previous PIs suggested, “*We need to persuade the community that data sharing helps everyone (including research participants) and improves the quality of science*”. Table 1 outlines specific actions and guidelines to improve data-sharing practices, focussing on researchers and research communities.

**Table 1:** Recommendations for improving data-sharing practices among researchers, institutions, publishers and funders in circadian mental health research.

Recommendation for researchers	
Use general repositories	Use platforms like GitHub and Zenodo for sharing data, especially in the absence of specialised repositories for specific data types.
Future repository linking	Instead of vaguely stating future data availability (data will be open), include a specific repository link in the data availability statement where data will be hosted, such as Zenodo’s versioning system or GitHub repositories, to ensure accessibility and proper version control.
FAIR training	Engage in training on FAIR principles and data management to improve data handling and sharing practices effectively.
Anonymise patient data	Implement data anonymisation to allow sharing sensitive patient data to maintain privacy and comply with ethical standards.
Ensure ethical data sharing	Seek ethical approval for data sharing to allow the sharing of full or partial datasets where possible, and secure appropriate participant consent.
Add a licence	Sharing data without a clear licence complicates reuse. Open licences (CC-0 or CC-BY) are recommended.
Share valuable metadata	Share metadata, research protocols and code, which are essential for replicability and transparency, even if data cannot be shared openly.
Use journal policies	Read the journal’s data-sharing policy and evaluate article compliance, as a reviewer or editor.
Recommendation for institutions	
Specialised training and curation services	Provide targeted training and data curation services to help researchers effectively manage and share data within specific research domains.
Institutional policies for data sharing	Develop policies that reward data sharing in recruitment and evaluation processes to encourage and recognise researchers who contribute to open data practices.
Best practices templates	Publish exemplary cases that illustrate successful integration of data-sharing practices within informed consent, data management and funding proposals to guide and motivate researchers.
Standardised README templates	Create and disseminate domain-specific README templates to ensure consistent and comprehensive data documentation across different research areas.
Recommendation for publishers and funders	
Integrate data verification in peer review	Evaluate the data-sharing statement and the data shared in the peer-review process for publication. This ensures that submitted data meet high standards of accessibility and usability.
Reward prior data-sharing efforts	Consider researchers’ previous data-sharing practices when allocating funds. Offer specific grants and funding opportunities to researchers who have demonstrated a strong commitment to open data practices.
Support for domain-specific repositories	Provide continuous funding and support for the development and maintenance of domain-specific data repositories. This will enable tailored data storage solutions that meet the unique requirements of circadian and mental health research data, as an example.

CMHN, Circadian Mental Health Network; DMP, data management plan ; FAIR, Findable, Accessible, Interoperable, Reusable; MRC, Medical Research Council; UKRI, UK Research and Innovation .

### Future and goals

As a CMHN, we plan to address the challenges of data sharing by delivering training on FAIR principles for data management, offering data curation services and providing draft consent forms or ethical guidelines for researchers. By implementing these practical strategies, we aim to support the research community in adopting better data-sharing practices. Additionally, further analysis of researchers’ beliefs and reasons to limit data sharing should distinguish between genuine concerns and misconceptions.^20^ We anticipate that in the next few years of these initiatives, there will be significant improvements in the sharing and accessibility of circadian and mental health research data.

## Data Availability

There is no new data generated from this perspective article. The data we discuss are in reference ^7^ and in addition, the ReadMe templates and guidelines that we refer to are openly available in a GitHub repository: https://github.com/circadianmentalhealth/circadian-data-standards
